# Accuracy and potential risk factors of pedicle screw placement using a noninvasive-registration robotic system in the thoracolumbar spine

**DOI:** 10.1007/s11701-025-02929-7

**Published:** 2025-11-11

**Authors:** Rui Yang, Kui Wang, Weilin Li, Jiajun Feng, Jian Jiang, Yuefeng Sun, Hong Wang

**Affiliations:** 1 Department of Orthopedics, The First Hospital of Lanzhou University, Lanzhou, Gansu Province China; 2 School of Graduates, Dalian Medical University, Dalian City, Liaoning Province China; 3 Department of Orthopedics, Central Hospital of Dalian University of Technology, Dalian City, Liaoning Province China

**Keywords:** Robot-Assisted spine surgery, ORTHBOT, Pedicle screw placement accuracy, Risk factors

## Abstract

To evaluate the accuracy of screw placement using the ORTHBOT robotic system in degenerative spine surgeries at our institution and identifying independent risk factors influencing screw deviation. The clinical data of 75 patients who underwent Robotic-Assisted Spine Surgery (RASS) at our hospital from May 1, 2022 to June 31, 2024. Concurrently, clinical data from 75 robot-assisted spine surgery cases in our treatment group were collected, including patients’ general information (age, gender, duration of disease, and length of hospital stay). Recorded parameters included the number of screws, operative time, BMI, bone density, degree of intervertebral disc degeneration (Pfirrmann grading), facet joint degeneration (Fuiiwara-MRI grading), vertebral rotation (Nash-Moe method), and screw grading (Gertzbein and Robbins scale). A total of 399 screws were evaluated. Statistical analysis was conducted using GraphPad software. Quantitative data were described according to their distribution, and categorical data were expressed as percentages. Initially, univariate analysis was performed, and variables with statistical significance were then incorporated into a multivariate logistic regression model to further assess the independent effects of each variable on screw placement accuracy. Screw placement accuracy was categorized as satisfactory or unsatisfactory, and the odds ratios (OR) with 95% confidence intervals (CI) for each influencing factor were calculated, with a *P* < 0.01 considered statistically significant. A total of 75 patients who underwent RASS surgery (all receiving percutaneous screw implantation) were included, comprising 40 males and 35 females, aged 25–84 years, with an average age of 63.81 ± 11.81 years. The patients had an average BMI of 25.74 ± 3.63, an average bone density T-score of − 0.19 ± 1.95, an average hospital stay of 13.15 ± 5.25 days, and an average disease duration of 50.88 ± 72.49 months. Among all patients, past medical histories included hypertension and diabetes, with 30 patients having hypertension and 7 having diabetes. All RASS procedures were performed under general anesthesia. A total of 329 Grade A screws (82.46%) and 32 Grade B screws (8.02%) were placed, with Grades A and B combined constituting satisfactory screws, totaling 361 (90.48%). Additionally, there were 26 Grade C screws (6.52%), 10 Grade D screws (2.51%), and 2 Grade E screws (0.50%), totaling 38 (9.52%), which were classified as unsatisfactory screws (Grades C, D, and E). Univariate analysis indicated that the risk of inaccurate screw placement was significantly higher in elderly patients (≥ 65 years) compared to the < 65 group, which is closely related to the common occurrence of osteoporosis, vertebral morphological variations, and unclear bony landmarks in the elderly. The risk was increased in patients with severe osteoporosis (T-score≤-3.5), suggesting that excessively low bone density reduces screw purchase, leading to screw trajectory deviation. BMI, disease duration, and operative time did not have a significant impact on the risk of unsatisfactory screw placement. Increased spinal rotation raised the risk (*P* = 0.009), reflecting the three-dimensional reconstruction challenge of pedicle spatial orientation posed by rotational deformities. An increased grade of facet joint degeneration had a significant impact (*P* < 0.05), possibly due to facet joint hypertrophy and sclerosis causing deviation of the K-wire from the planned trajectory. Multivariate logistic regression analysis indicated that severe vertebral rotation (Grade III–IV) (*P* < 0.01) significantly affected screw accuracy, as spinal rotation limited the range of motion of the robotic arm. In osteoporotic patients, decreased bone mineral density (BMD) significantly increased the risk of failure, with an odds ratio (OR) of 1.832 [95% CI: 1.212–2.741] when-3.5 < T≤-2.5, and an OR of 3.502 [95%CI: 1.923–6.384] when T≤-3.5. This may be related to lower screw purchase and screw displacement during decompression surgery. A facet joint degeneration grade higher than 2 also increased the risk of screw displacement (*P* < 0.01), as more severe degeneration, with increased osteophyte formation and higher cortical bone density, may increase the difficulty of K-wire insertion, leading to displacement on the facet joint surface. Age did not have a significant impact on screw accuracy (*P* = 0.028). This study conducted a retrospective analysis of clinical data from 75 patients undergoing robot-assisted spinal surgery (RASS), confirming the high precision of the domestically developed ORTHBOT system in pedicle screw placement, with a satisfactory screw (Grade A/B) rate of 90.48%. These findings provide reliable evidence for the clinical application of RASS technology in degenerative spinal diseases. Univariate analysis identified age, spinal rotation grade, bone mineral density (BMD), and facet joint degeneration grade as risk factors leading to reduced screw placement accuracy, while BMI, disease duration, and operative time showed no significant influence. Multivariate regression analysis further determined that bone density loss (T-score≤-2.5), grade III–IV spinal rotation, and grade 2–3 facet joint degeneration were independent risk factors for screw deviation.

## Introduction

The accuracy of pedicle screw placement directly impacts the outcomes of spinal surgery. Traditional freehand or percutaneous screw insertion, which heavily relies on the surgeon’s experience, carries risks of screw deviation and may lead to complications such as nerve injury or implant failure [1]. Robot-assisted spinal surgery (RASS) offers a novel solution by combining three-dimensional image navigation with robotic arm trajectory guidance technology. Previous systems such as Mazor X and ROSA Spine have demonstrated clinical value in minimally invasive procedures [2], and literature reports consistently indicate higher satisfaction rates for RASS screws compared to freehand or percutaneous techniques [3–5]. However, risks of pedicle screw misplacement remain, and the current risk assessment system for the impact of spinal degeneration on screw accuracy remains inadequate [6].

Our institution has introduced ORTHBOT, a domestically developed next-generation orthopedic robotic platform designed to enhance the safety and precision of spinal instrumentation. Unlike existing robotic systems that require invasive placement of a reference frame—such as a sacral reference clamp—to register the patient’s anatomy with preoperative images, ORTHBOT employs a noninvasive dynamic binocular registration module. In this system, a positioning plate with a distinctive visual pattern is placed above the surgical field. The binocular vision module mounted on the robotic arm performs real-time spatial calibration of the arm’s position, while intraoperative fluoroscopy of the positioning plate and operative region enables automatic registration with preoperative imaging. This process eliminates the need for invasive reference frame installation without compromising registration accuracy, thereby simplifying the workflow and minimizing soft tissue disruption. In addition, the system incorporates a cortical protection through real-time pressure monitoring, which further improve screw trajectory accuracy and intraoperative safety. Nevertheless, its accuracy and associated risk factors require further validation.

We conducted a statistical analysis of RASS cases from the past two years to evaluate the screw placement accuracy and risk factors of this new robotic system. By comparing our data with existing domestic and international literature, we aimed to assess the current state of RASS technology in our hospital, identify areas for improvement, and define future directions for our discipline. Additionally, we hope to provide valuable insights and references for the broader medical community.

## Materials and methods

### Data collection

This study employed a retrospective descriptive research method, enrolling patients who underwent robot-assisted spinal surgery (RASS) at the Affiliated Central Hospital of Dalian University of Technology between May 1, 2022, and June 30, 2024. Based on the aforementioned inclusion criteria, a total of 75 patients were included in this study, comprising 40 males and 35 females, with an age range of 25–84 years (mean age: 63.81 ± 11.81 years). All clinical data were collected by the same surgeon, and the surgical procedures were performed by a single experienced orthopedic surgeon using the ORTHBOT integrated powered orthopedic robotic system.

Patient demographics and clinical parameters were systematically collected. To facilitate data analysis, the clinical variables were categorized as follows:

1. General characteristics: age, gender, disease duration, and length of hospital stay;
2. Surgical parameters: number of screws implanted and operative time (from anesthesia induction to procedure completion);
3. Physiological metrics: body mass index (BMI) and bone mineral density (measured by dual-energy X-ray absorptiometry, DXA);
4. Degenerative assessments:

Intervertebral disc degeneration (Pfirrmann grading system).

Facet joint degeneration (Fujiwara-MRI classification).

Vertebral rotation (Nash-Moe method).

5. Screw placement accuracy (Gertzbein and Robbins classification).

### Surgical procedure

Preoperative Planning and Workstation Setup: The thin-slice CT scan data in DICOM format were imported into the ORTHBOT robotic system workstation for 3D reconstruction. Based on the reconstructed anatomy, the optimal position and size of K-wires or screws were planned and verified by the same surgeon. Considering the degree of spinal degeneration, the surgical plan was adjusted to determine the appropriate screw dimensions and optimal trajectory in the coronal, sagittal, and axial planes. After anesthesia induction, the patient was placed in the prone position, and the workstation was connected to the C-arm X-ray machine and the orthopedic surgical robot, ensuring the robotic arm operated within a safe range.

Intraoperative Registration: The C-arm fluoroscopic images were matched with the preoperative CT data, and the surgical plan was uploaded to the workstation via a local network after registration. The robotic arm was aligned with the patient’s position, and the operator assembled the reference frame and positioned it above the surgical site. Anteroposterior (AP) and lateral fluoroscopic images were acquired using the C-arm and registered with the preoperative CT data to complete the matching process.

K-Wire Guidance and Adjustment: The binocular recognition system identified markers on the reference frame to guide the robotic arm to the target surgical site. The K-wire was positioned, a skin incision was made, and the cannula was advanced to the cortical bone. The robot automatically activated the drill according to the preoperative plan to insert the K-wire. Intraoperative lateral fluoroscopy was used to verify placement, and K-wire insertion pressure was monitored to ensure safety. The surgeon determined whether adjustments were needed based on experience. After final positioning, AP and lateral fluoroscopic images were obtained for confirmation. Once all K-wires were placed, the robotic arm was removed from the surgical field.

Pedicle Screw Insertion: Following decompression, the surgeon proceeded with pedicle screw placement.

### Statistical analysis

Statistical analysis was performed using GraphPad software. Continuous variables conforming to normal distribution were expressed as mean ± standard deviation (SD), while non-normally distributed data were presented as median (interquartile range, IQR). Categorical variables were expressed as percentages.

Univariate analysis was first conducted to screen potential factors affecting screw placement accuracy. For continuous variables (age, BMI, T-score, disease duration), independent samples t-test was used for normally distributed data, while Mann-Whitney U test was employed for non-normally distributed data. For categorical variables (facet joint degeneration grade, intervertebral disc degeneration grade, vertebral rotation grade), Chi-square test was applied, with Fisher’s exact test being used when sample sizes were small.

Variables demonstrating statistical significance (*P* < 0.05) in univariate analysis were subsequently incorporated into logistic regression models to evaluate their independent effects on screw placement accuracy. The dependent variable was defined as screw accuracy grouping (satisfactory/unsatisfactory). Odds ratios (OR) with 95% confidence intervals (CI) were calculated for each influencing factor, with *P* < 0.01 set as the threshold for statistical significance.

## Results

### Overview

**Fig. 1:**
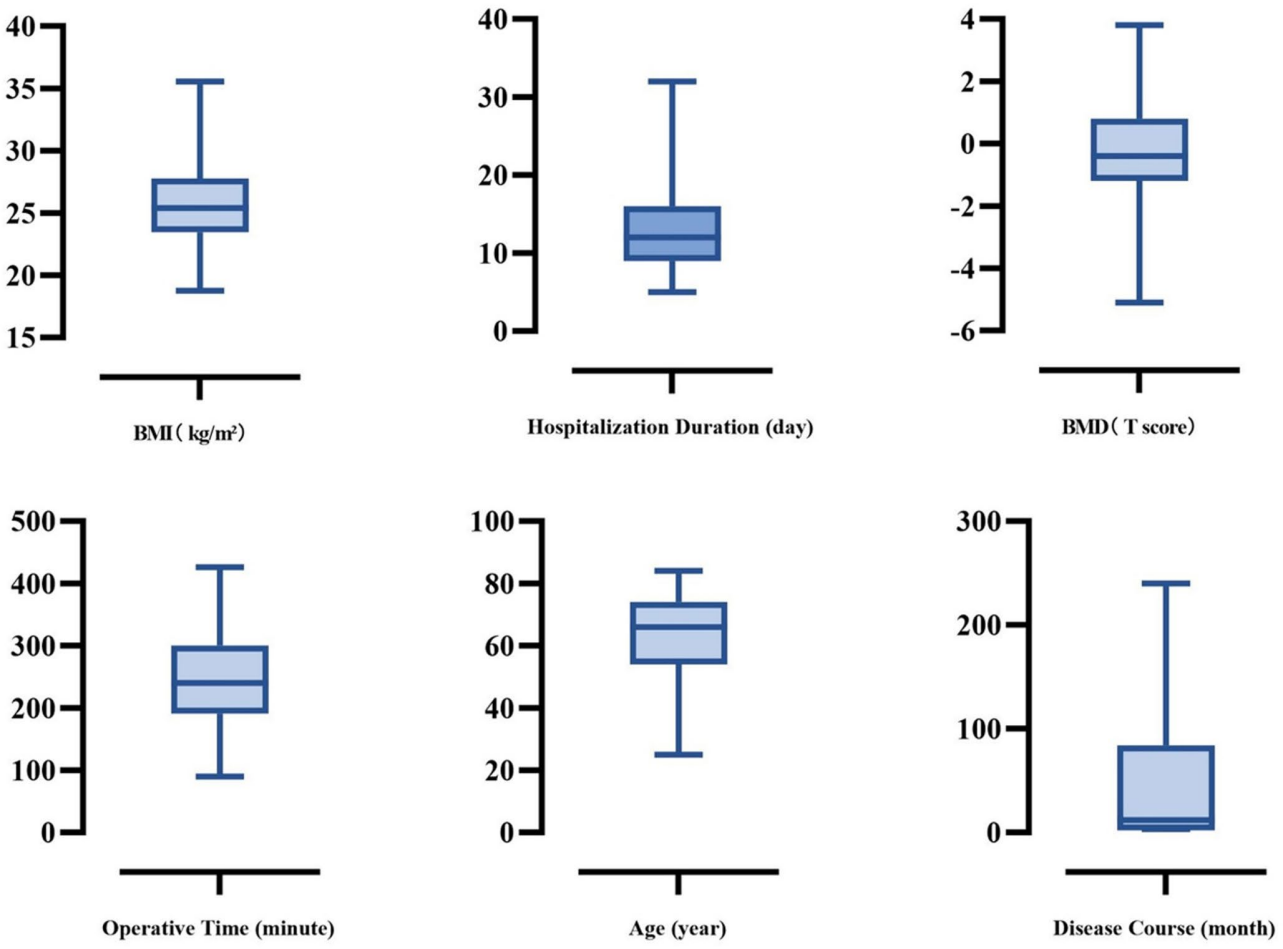
Patient general clinic data

This study included 75 patients who underwent robot-assisted spinal surgery (RASS) with percutaneous screw placement, comprising 40 males and 35 females aged 25–84 years (mean age: 63.81 ± 11.81 years; Fig. 1). The cohort demonstrated a mean BMI of 25.74 ± 3.63 and an average bone mineral density T-score of −0.19 ± 1.95 (two younger patients without T-score measurements were excluded from bone density analysis). The mean hospitalization duration was 13.15 ± 5.25 days, with an average disease course of 50.88 ± 72.49 months.

Among all participants, 30 patients had pre-existing hypertension and 7 had diabetes mellitus as underlying comorbidities. All procedures were performed under general anesthesia, with both surgical execution and planning verification conducted by the same surgeon to ensure procedural consistency.

In this study, the parameters related to spinal degeneration were as follows (Table 1): mean facet joint degeneration grade (Fujiwara-MRI classification) was 1.84 ± 1.07; mean intervertebral disc degeneration grade (Pfirrmann classification) was 3.99 ± 1.01; and mean vertebral rotation (Nash-Moe method) was 0.91 ± 0.95. Regarding surgical parameters (Table 2), the average number of K-wires used was 5.32 ± 1.44, and the mean operative time was 241.92 ± 70.03 min.

**Table 1:** Degeneration of spinal units and pedicle screw classification

Intervertebral Disc Degeneration Grading						
Intervertebral Disc Degeneration Grading (Pfirrmann Classification)		Ⅰ	Ⅱ	Ⅲ	Ⅳ	Ⅴ
Number of Patients		0	8	14	24	29
Facet Joint Degeneration Grading (Fujiwara-MRI Classification)		0		1	2	3
Number of Patients		12		13	25	25
Spinal Rotation Grading (Nash–Moe Method)		0	Ⅰ	Ⅱ	Ⅲ	Ⅳ
Number of Patients		31	26	12	6	0
Pedicle Screw Grading (Gertzbein and Robbins scale)						
A	B	C		D	E	
329	32	26		10	2	
Satisfied Group				Dissatisfied Group		
361(90.48%)				38(9.52%)		
Total Screws: 399						

**Table 2:** Analysis of single-factor spinal degeneration and pedicle screw grading

Variable	Satisfied Group	Dissatisfied Group	*P* value^*^
Age (years)			
<65	147	16	<0.05
≥65	214	22	
Gender (Male/Female)	202/160	17/20	0.286
BMI (kg/m^2^ )			
<27	264	32	0.263
≥27	98	5	
Spinal Rotation Grading (Nash–Moe Method)			
0	137	19	<0.05
I-II	192	17	
III-IV	32	2	
BMD (T score)			
>−2.5	305	29	<0.05
−3.5 <T≤−2.5	34	5	
T≤−3.5	10	4	
Facet Joint Degeneration Grading (Fujiwara-MRI Classification)			
0	50	6	<0.05
1	55	6	
2	127	13	
3	126	13	
Intervertebral Disc Degeneration Grading (Pfirrmann Classification)			
I-II	48	0	0.450
III	63	7	
IV-V	251	30	
Duration of Disease (months)	(50.88±72.49)		0.828
Operation Time (minutes)	(241.92±70.03)		0.355

Postoperative radiographic evaluation assessed a total of 399 implanted screws, all graded for accuracy using the Gertzbein and Robbins classification system. This system categorizes screws into five grades:

#### Grade A

Screw completely contained within the pedicle cortex without breach;

#### Grade B/C/D

Screw breaching the pedicle cortex by < 2 mm, < 4 mm, and < 6 mm, respectively;

#### Grade E

Screw deviating > 6 mm from the pedicle or completely misplaced outside the pedicle.

Among the evaluated screws, 329 (82.46%) were Grade A and 32 (8.02%) were Grade B, collectively constituting satisfactory placements (Grades A + B, totaling 90.48%). The remaining 38 screws (9.52%) were classified as unsatisfactory: 26 (6.52%) Grade C, 10 (2.51%) Grade D, and 2 (0.50%) Grade E.

### Univariate analysis

Variables were optimally categorized to enhance the interpretability of their association with screw placement accuracy.

### Continuous variables

#### Age

Stratified into < 65 years and ≥ 65 years groups.

#### BMI

Grouped using Asian obesity criteria (≥ 27 kg/m²) to assess potential interference with instrumentation.

#### Bone mineral density (BMD, T-score)

Classified per WHO osteoporosis criteria into T > −2.5, −3.5 < T ≤ −2.5, and T ≤ −3.5, reflecting the gradient effect of reduced BMD on screw anchorage. (One young patient without BMD data was excluded from this analysis.)

### Categorical variables

#### Facet joint degeneration

Fujiwara-MRI grading (0–3) evaluated osteophyte impact on screw stability.

#### Vertebral rotation

Nash-Moe grading (0–IV) quantified rotational deformity’s effect on pedicle 3D morphology.

#### Disc degeneration

Pfirrmann grading (I–V) assessed disc collapse and signal changes. After categorization, Shapiro-Wilk test confirmed normality. Non-normally distributed variables were analyzed via Mann-Whitney U test, while categorical variables used Chi-square/Fisher’s exact tests.

**Key findings**:

1. Older patients (≥ 65 years) had significantly higher screw inaccuracy risks (*P* < 0.05), likely due to osteoporosis, vertebral morphological variations, and obscured bony landmarks.
2. Severely reduced BMD (T ≤ −3.5) increased risk, suggesting poor screw purchase and trajectory deviation.
3. BMI, disease duration, and operative time showed no significant impact.
4. Vertebral rotation (*P* = 0.009) elevated inaccuracy risk, highlighting 3D reconstruction challenges in rotated pedicles.
5. Advanced facet joint degeneration (*P* < 0.05) correlated with K-wire deviation, possibly due to osteosclerosis altering planned trajectories.

### Multivariate logistic regression

Before modeling, multicollinearity was assessed using variance inflation factors (VIFs): Age (VIF = 1.5), BMD (VIF = 1.7), facet degeneration (VIF = 1.6), vertebral rotation (VIF = 1.9). All VIFs < 3 indicated negligible collinearity, ensuring stable independent variable estimates.

Significant univariate predictors (age, BMD, facet degeneration, vertebral rotation) were included in the multivariate logistic regression, with screw accuracy (satisfactory/unsatisfactory) as the outcome (Table 3).

**Table 3:** Analysis of multi-factor logistic regression of spinal degeneration and pedicle screw grading

Independent Variable	OR (95% CI)	P值
Spinal Rotation Grading (Nash–Moe Method)		
III-IV	4.231(2.123–8.501)	<0.01
BMD (T score)		
−3.5 < T ≤ −2.5	1.832(1.212–2.741)	<0.01
T ≤−3.5	3.502(1.923–6.384)	<0.01
Facet Joint Degeneration Grading (Fujiwara-MRI Classification)		
2–3	2.145(1.312–3.432)	<0.01
Age (years)		
≥65	2.307(1.123–4.802)	0.028

1. Severe vertebral rotation (Grade III–IV):

*P* < 0.01; OR not calculated (reference).

Mechanistic insight: Rotation limits robotic arm maneuverability.

2. Reduced BMD:

−3.5 < T ≤ −2.5: OR 1.832 [95% CI 1.212–2.741].

T ≤ −3.5: OR 3.502 [95% CI 1.923–6.384].

Proposed mechanism: Poor screw purchase post-decompression increases displacement risk.

3. Facet degeneration (Grade ≥ 2):

*P* < 0.01; OR not reported.

Explanation: Osteophytes and uneven cortical density may deflect K-wires during insertion.

4. Age:

*P* = 0.028 (non-significant in multivariate analysis).

## Discussion

### The development of robot-assisted spinal surgery (RASS)

Robot-assisted spine surgery (RASS) has undergone a significant evolution since its initial application in spinal surgery in the 1990 s, transitioning from basic exploratory stages to a mature and widely adopted technique. With continuous technological advancements, RASS has evolved from a simple robotic arm system into a multifunctional and highly precise tool for spinal surgical assistance [7]. In spinal procedures, the placement of pedicle screws is crucial, serving to stabilize the spinal structure. This technique is extensively used in the treatment of spinal deformities, fractures, degenerative diseases, and tumors, representing a major milestone in the development of spinal surgery [8]. However, due to the proximity of the pedicle to nerve roots, the spinal cord, and blood vessels, screw placement carries certain risks and potential complications. Therefore, improving the accuracy of screw placement has always been a critical goal in pedicle screw fixation techniques [9].

With the advancement of medical imaging and computer technologies, image navigation and robotic systems have gradually been applied to pedicle screw placement. Renaissance was the first robotic system approved by the FDA for use in spinal surgery [10], and systems such as SpineAssist and Mazor X have garnered widespread global attention and application [11–14]. Studies have shown that robot-assisted surgery can enhance screw accuracy, reduce radiation exposure, and lower complication rates. However, some literature has reported inconsistent performance in robotic screw placement accuracy. Even prospective randomized controlled trials and meta-analyses have indicated that robotic-assisted placement may not always outperform manual or percutaneous screw placement [15]. These discrepancies may be attributed to factors such as disease type, patient-specific differences, and variations in surgical protocols. It is noteworthy that most spinal surgery robots are semi-autonomous, with many operative steps still requiring manual execution by surgeons, suggesting that multiple factors in clinical practice may influence placement accuracy.

Currently, most spinal surgery robots focus primarily on guiding the trajectory of pedicle screws, assisting surgeons in identifying the optimal insertion path. However, the actual insertion of K-wires and screws remains manual [16]. Addressing this limitation, the ORTHBOT system is the first to achieve automated K-wire insertion, surpassing the traditional navigation-only capability [5]. Its pressure monitoring and cortical wall protection function enables real-time surveillance of pressure changes during insertion, simulating tactile feedback and stopping automatically when abnormal conditions are detected, thereby preventing cortical breach. Furthermore, ORTHBOT is equipped with a binocular visible light navigation system for close-range surgical monitoring, reducing external interference and improving operational efficiency. Its non-invasive, contact-free localization technology avoids the need for invasive bone-mounted markers, significantly reducing surgical trauma to patients [3]. Additionally, its multimodal fusion of preoperative and intraoperative imaging shortens intraoperative planning time and minimizes radiation exposure for both surgeons and patients, offering enhanced precision and safety in spinal surgery.

Numerous studies have demonstrated that RASS presents a relatively short learning curve for pedicle screw placement. Shi and Chen retrospectively analyzed 95 patients (541 screws) who underwent robot-assisted spinal surgery using the ExcelsiusGPS system to assess the learning curve and proficiency development. The results showed that total operative time significantly decreased after approximately 14 cases, and screw insertion time improved after about 13 cases, indicating that surgeons can rapidly acquire key skills for robot-assisted surgery. Interestingly, robot registration time remained relatively unchanged, and the trends persisted even after controlling for fusion segment number, supporting the notion of a short learning curve and rapid skill acquisition, which bolsters the clinical adoption of RASS. Feng and Fan conducted a comparative cohort study involving 199 patients who underwent posterior pedicle screw fixation, with two junior surgeons performing screw insertions under senior expert supervision. A total of 769 screws were inserted in the robot group and 788 in the manual group. The study found that in the upper thoracic segment, the robot group had a shorter learning phase, while longer learning periods were observed in the lower thoracic and lumbosacral segments. Overall, the robot group exhibited a gentler learning curve slope, and the accuracy of screw placement in the upper thoracic region was significantly higher in the robot group (89.4% vs. 76.7%, *P* < 0.001), with more pronounced differences in deformity cases. Although the robot group had slightly shorter screw placement times in the upper thoracic region, the manual group performed faster in lower thoracic and lumbosacral regions, with three screw-related neurological complications occurring exclusively in the manual group. These results suggest that robotic assistance offers significant advantages in the upper thoracic region and in cases of deformity, allowing junior surgeons to quickly grasp critical skills and achieve high accuracy and safety even in early adoption stages. Whether utilizing specialized navigation systems like ExcelsiusGPS^®^ or targeting specific procedures such as percutaneous pedicle screw placement or vertebroplasty [17], surgeons can significantly shorten operation time and master robot registration and insertion techniques after roughly a dozen cases. Some literature has emphasized the consistent superiority of robotic screw placement in upper thoracic and structurally deformed cases [18], even in early use. With increasing experience and proficiency, overall operation time, radiation exposure, and complication rates in robot-assisted surgery can be effectively maintained at low levels [19–22], providing further support for the clinical implementation of RASS.

RASS not only assists with pedicle screw placement but is also widely applicable in the comprehensive treatment of spinal deformities, tumor resection, fracture repair, and degenerative diseases [23]. Despite challenges such as high equipment costs and complex operations, RASS is increasingly becoming a key component of spinal surgery as the technology matures and gains acceptance in major healthcare institutions.

### Risk factors for pedicle screw misplacement

The accuracy of pedicle screw placement is one of the key determinants of success in spinal surgery. Misplacement of screws can lead to postoperative complications such as thrombosis, fixation failure, and chronic pain. Even in RASS, the risk of screw deviation remains a concern [24–27]. Studies have shown that osteoporosis (especially with T-scores below − 3.5), high degrees of spinal rotation (Nash-Moe grade ≥ II), and severe facet joint degeneration (Fujiiwara-MRI grade ≥ III) all significantly increase the likelihood of cortical breach and facet joint violation [1, 25, 28]. In this study, two patients with Grade E screws had all three of the above high-risk factors, suggesting that degenerative spines and reduced bone density pose significant challenges to the navigation accuracy of RASS [29]. Additionally, for severely obese patients, those with osteoporosis, or cases requiring screw placement more than three segments away from the reference point, surgeons must exercise heightened caution to avoid postoperative complications due to unsatisfactory screw positions [3].

From a clinical translation perspective, our analysis of risk factors highlights the necessity of implementing precision strategies for specific patient populations. For patients who are elderly (≥ 65 years), have low bone density (T≤−2.5), spinal rotation ≥ Grade III, or facet joint degeneration ≥ Grade III, it is essential to enhance preoperative bone density assessment and quantify spinal rotation. For T-scores ≤−3.5, preoperative planning for cement augmentation or using cortical screws may improve screw anchorage. This should be combined with intraoperative image guidance and manual adjustments. Furthermore, robotic systems should be optimized to address issues such as thick soft tissue and positioning changes by developing specialized puncture channels and improving registration algorithms.

Although RASS can utilize 3D imaging and robotic navigation to reduce errors, it still largely depends on manual execution by the surgeon. Therefore, reducing placement errors in high-risk populations has become a new research focus. The ORTHBOT system used in this study incorporates intelligent needle insertion and pressure monitoring cortical protection features, aiming to address complex conditions such as spinal degeneration and osteoporosis more effectively than existing systems. For example, its real-time pressure monitoring allows the system to shut down promptly when encountering abnormal resistance in cartilaginous or osteoporotic bone, minimizing potential injury to surrounding tissues. Multimodal image fusion also eliminates the need for invasive reference frames. This study analyzes the accuracy and risk factors of the ORTHBOT system in clinical use based on this design philosophy.

### Future directions for RASS

RASS is expected to see broader development and application in the future. On the one hand, the rise of artificial intelligence and machine learning has led to a surge in studies focused on surgical prediction and automatic image recognition in spine surgery. AI algorithms can perform deep learning on vast preoperative and intraoperative imaging datasets, offering personalized recommendations for disease type, surgical approach, and screw sizing and trajectory, as well as real-time optimization based on anatomical and pathological variations. This approach provides more accurate and efficient decision support in patient selection, surgical planning, and postoperative recovery, reducing overtreatment and complications to achieve better clinical outcomes [30]. On the other hand, mixed reality (MR) technologies are rapidly emerging in spine and orthopedic surgery. Studies show that head-mounted augmented reality devices can achieve comparable precision to robotic systems in pedicle screw navigation [31–37]. During surgery, AR technology overlays digital images onto real anatomical views, allowing real-time visualization of spinal structures, blood vessels, and nerve pathways, enhancing targeting precision, reducing radiation exposure time, and alleviating surgeon fatigue [34, 38].

Importantly, the application of robotic technology in spine surgery extends beyond pedicle screw insertion. Bibliometric analysis of research trends indicates emerging focus on cortical bone trajectory screws. Robotic systems have also been used in laminectomy, spinal canal decompression, spinal tumor resection, and various endoscopic procedures to enhance precision and safety [39, 40]. Some new robotic systems have been applied in multi-level lumbar fusions, scoliosis correction, and complex spinal reconstruction surgeries [16, 41]. Others have explored their use in accurate puncture and injection during vertebroplasty [31]. Robot assistance has also shown value in endoscopic surgery. A prospective cohort study compared percutaneous endoscopic robot-assisted transforaminal lumbar interbody fusion (PE RA-TLIF) with traditional minimally invasive TLIF (MIS-TLIF) in patients with L4-5 spondylolisthesis. The study included 58 patients, with 26 undergoing PE RA-TLIF using robotic-guided screw insertion, full endoscopic decompression, and interbody fusion, while the MIS-TLIF group used conventional techniques. Results showed early postoperative advantages for the PE RA-TLIF group, such as smaller incisions, reduced blood loss, and less incision-related pain. Although this group had longer operation times, duration decreased with experience. Both groups showed no significant differences in long-term outcomes, including JOA scores, disability index, leg pain relief, and fusion rates, but the PE RA-TLIF group had better low back pain relief. The authors concluded that PE RA-TLIF enhances screw accuracy, reduces surgical trauma, and supports faster recovery, though it presents a steep learning curve and requires long-term follow-up to confirm lasting efficacy. Robotic systems also show promise in other orthopedic subspecialties. For example, in knee ligament reconstruction, smart planning of tunnel angles and depths can reduce overlap or collisions, lowering intraoperative errors and postoperative complications [42]. With continued upgrades in hardware and surgical workflow, new-generation spinal robots are expanding to broader indications. Studies have shown robotic systems to be safe and feasible for laminectomy [40], and systems like TiRobot offer superior accuracy and fewer complications in percutaneous pedicle screw placement for thoracolumbar fractures compared to conventional fluoroscopy [43]. RASS also yields satisfactory imaging and clinical outcomes in elderly osteoporotic patients undergoing cement augmentation, adolescent idiopathic scoliosis (AIS) correction, and spine trauma surgeries [19, 43–45]. Current research suggests no significant differences in blood loss and complication rates between RASS and traditional techniques, but robot-assisted surgery offers potential advantages in reducing surgeon fatigue, streamlining workflows, and enhancing screw or drill positioning accuracy [43]. As costs decline and surgical team training improves, RASS is expected to become more widely adopted, especially in resource-limited regions where it may enhance surgical quality and reduce error rates [45].

In addition to technical and clinical advancements, future studies should also focus on the economic implications of robotic systems. A comprehensive cost-effectiveness analysis comparing ORTHBOT with other mainstream robotic platforms (such as Mazor X, ROSA Spine, and TiRobot) would provide valuable insights into the balance between surgical efficiency, clinical outcomes, and healthcare expenditure. Such evidence would further clarify the clinical and economic utility of ORTHBOT and support its broader adoption in spine surgery practice.

Overall, the integration of advanced technology with robotic systems has created new possibilities for spine and broader orthopedic surgery. Continued expansion in procedures such as deformity correction, laminectomy, and endoscopic decompression is making surgery increasingly personalized, precise, and minimally invasive. Meanwhile, robotic and navigation technologies are extending into joint surgery. With the advancement of randomized controlled trials and multicenter studies, the next generation of RASS will become more refined, enriching its indications and safety evaluation frameworks, and playing an increasingly important role in orthopedic clinical decision-making and surgical operations.

## Conclusions

This study retrospectively analyzed clinical data from 75 patients who underwent robot-assisted spine surgery (RASS), confirming the high accuracy of the domestic ORTHBOT system in pedicle screw placement, with a satisfactory screw rate (Grade A/B) of 90.48%, providing reliable support for the clinical application of RASS in degenerative spinal diseases. In univariate analysis, aging, decreased bone density, spinal rotation, and facet joint degeneration were identified as risk factors for reduced screw accuracy. Intervertebral disc degeneration grade, BMI, disease duration, and operative time had no significant impact on screw accuracy. Multivariate analysis identified decreased bone density (T≤−2.5), Grade III–IV spinal rotation, and Grade 2–3 facet joint degeneration as independent risk factors for reduced screw accuracy, while aging alone was not statistically significant.

### Limitations

This study has several limitations. First, the retrospective single-center design may introduce selection bias, and the overall sample size is relatively small (*n* = 75), particularly in the unsatisfactory screw group (grades C/D/E), which included only 38 screws. The low number of Grade D and E screws may limit the statistical power of both univariate and multivariate analyses, and some identified associations could be due to chance rather than a true effect. Second, the lack of long-term follow-up data, such as screw loosening rates and clinical functional scores at 1 year postoperatively, restricts a comprehensive evaluation of the relationship between screw accuracy and prognosis. Third, this study did not include a comparative analysis with freehand or fluoroscopy-guided percutaneous screw placement, limiting the assessment of relative performance. Fourth, bone mineral density (BMD) measurements were unavailable for some younger patients (under 30 years old), and the corresponding screws (all within the satisfactory group) were excluded from the univariate BMD analysis, which may affect the precision of the results. Finally, early cases of robot-assisted surgery performed by the surgical team were not excluded, so the screw placement accuracy may have been influenced by the learning curve. Future prospective multicenter studies with larger sample sizes are warranted to further validate these findings and assess their generalizability.

## Data Availability

All data analyzed was included in this study, further request could be consulted and obtained from correspondent author.

## Declarations

### **Declaration of AI and AI-assisted technologies in the writing process**

We declared that no AI and AI-assisted technologies used during writing this paper.
